# 3D respiratory resolved phase contrast imaging of the aorta

**DOI:** 10.1186/1532-429X-15-S1-P250

**Published:** 2013-01-30

**Authors:** Eric M Schrauben, Ashley G Anderson, Kevin M Johnson, Oliver Wieben

**Affiliations:** 1 Medical Physics, University of Wisconsin - Madison, Madison, WI, USA

## Background

Respiratory motion compensation is essential for reproducible and robust cardiovascular MRI. Traditionally, breathholds or prospective gating by bellows or navigator signals limit data acquisition to the quiescent phase of respiration [1]. These approaches do not capture any variations of blood flow over the respiratory cycle, yet respiration has been shown to significantly affect flow in the great vessels [2]. The purpose of this pilot study was to adapt a 3D radially undersampled PC MR sequence (PC VIPR [3,4]) for use with our retrospective dual-gated (cardiac and respiratory) reconstruction to evaluate respiratory effects on net flow and cardiac flow waveforms.

## Methods

**Figure 1:**
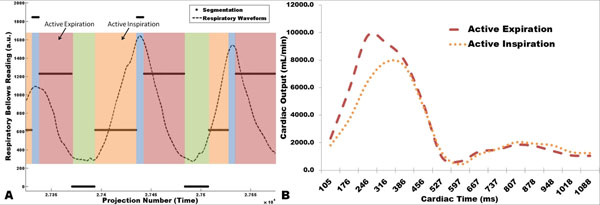
Example calculation of respiratory phase position using the respiratory waveform (a), which can be sorted in any fashion based on the temporal feature the user wishes to investigate. Flow analysis shows increased flow during active expiration versus active inspiration (b). Volunteer cardiac signal was acquired with a pulse oximeter.

Five healthy volunteers were imaged on a 3.0T system (Discovery MR750, GE, WI) using PC VIPR prescribed over a large chest imaging volume (FOV = 32 x 32 x 32 cm3, time resolution = 55 ms, TR/TE = 6.3/2.1 ms, α = 10°, Venc = 140-150 cm/s, projection number ≈ 44000, 16 cardiac timeframes). Interleaving of radial projections that traverse the center of k-space allow for retrospective sorting in a flexible fashion. Our traditional retrospective ECG gating was expanded to incorporate additional sorting of the data into respiratory phases based on the bellows signal (Fig. 1a) to provide cardiac series corresponding to separate respiratory phases. The phases used were active inspiration and expiration, and their two corresponding plateaus. Cardiac timeframes are reconstructed using temporal view sharing to improve the image quality by reducing undersampling artifacts [5]. This process results in a dual-retrospectively-gated PC-MR exam. Data were continuously acquired during free breathing in contrast to prospectively gated chest PC-MR exams that discard 50-60% of data. Thus total scan time was unchanged. Flow analysis was performed in the ascending and descending aorta (AAo, DAo).

## Results

**Figure 2:**
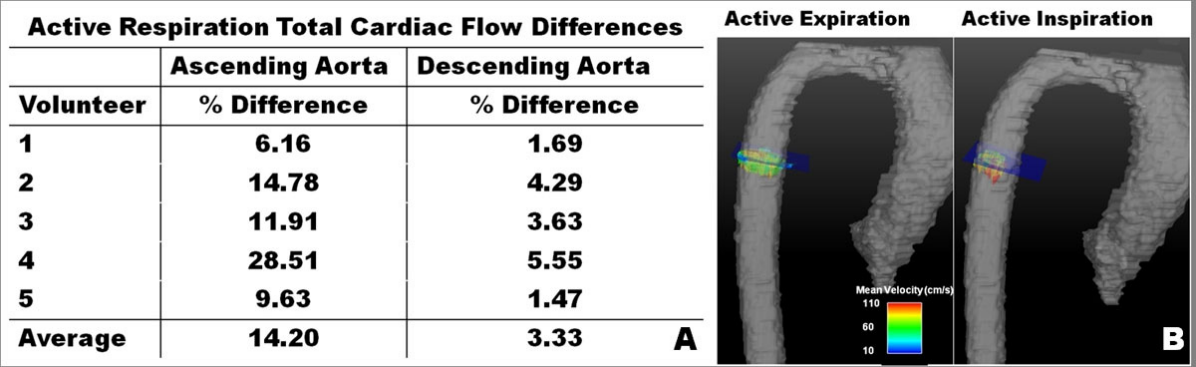
Average percent difference between active inspiration and active expiration of instantaneous flow (a) over all volunteers in both the ascending and descending aorta. 3D vector visualizations (b) in the descending aorta shows similar respiratory flow features.

Representative flow waveforms for the DAo during inspiration and expiration are shown in Figure 1b. Figure 2a displays average% difference of flow over the cardiac cycle for each of the five volunteers comparing active inspiration and expiration. In all five cases, expiration increased both AAo and DAo flow compared to inspiration. Figure 2b shows 3D vector visualizations of the two active respiration reconstructions at the measurement plane for the DAo.

## Conclusions

A 3D radial double-gated acquisition and reconstruction that allows for blood flow analysis over the cardiac cycle is presented. Data acquired in healthy volunteers shows significant increased blood flow during expiration compared to inspiration in the Ao. The same effect is marginal in the DAo. Acquisition throughout the respiratory cycle does not prolong scan time while allowing for retrospective selection of arbitrary measurement planes. Further studies are warranted to quantify these respiratory effects in other vessels and to investigate implications for assessing flow.

## Funding

NMSS fund RC1003-A-1, NIH grant 2R01HL072260, and GE Healthcare
